# Immunogenicity and protective efficacy of GBP510/AS03 vaccine against SARS-CoV-2 delta challenge in rhesus macaques

**DOI:** 10.1038/s41541-023-00622-0

**Published:** 2023-02-23

**Authors:** Catherine Jacob-Dolan, Jingyou Yu, Katherine McMahan, Victoria Giffin, Abishek Chandrashekar, Amanda J. Martinot, Tochi Anioke, Olivia C. Powers, Kevin Hall, David Hope, Jessica Miller, Nichole P. Hachmann, Benjamin Chung, Sarah Gardner, Daniel Sellers, Julia Barrett, Mark G. Lewis, Hanne Andersen, Harry Kleanthous, Ki-Woen Seo, Su Jeen Lee, Yong Wook Park, Hun Kim, Dan H. Barouch

**Affiliations:** 1 Center for Virology and Vaccine Research, Beth Israel Deaconess Medical Center, Boston, MA 02215 USA; 2 Ragon Institute of MGH, MIT and Harvard, Cambridge, MA USA; 3 Harvard Medical School, Boston, MA 02115 USA; 4 Tufts Cummings School of Veterinary Medicine, North Grafton, MA 01536 USA; 5 Bioqual, Rockville, MD 20852 USA; 6 Bill and Melinda Gates Foundation, Seattle, WA 98109 USA; 7 Department of Research and Development, SK bioscience 310 Pangyo-ro, Bundang-gu, Seongnam-si, Gyeonggi-do Republic of Korea

**Keywords:** Protein vaccines, Protein vaccines

## Abstract

Despite the availability of several effective SARS-CoV-2 vaccines, additional vaccines will be required for optimal global vaccination. In this study, we investigate the immunogenicity and protective efficacy of the GBP510 protein subunit vaccine adjuvanted with AS03, which has recently been authorized for marketing in South Korea under the trade name SKYCovione^TM^. The antigen in GBP510/AS03 is a two-part recombinant nanoparticle, which displays 60 receptor binding domain (RBD) proteins of SARS-CoV-2 Spike on its surface. In this study we show that GBP510/AS03 induced robust immune responses in rhesus macaques and protected against a high-dose SARS-CoV-2 Delta challenge. We vaccinated macaques with two or three doses of GBP510/AS03 matched to the ancestral Wuhan strain of SARS-CoV-2 or with two doses of GBP510/AS03 matched to the ancestral strain and one dose matched to the Beta strain. Following the challenge with Delta, the vaccinated macaques rapidly controlled the virus in bronchoalveolar lavage and nasal swabs. Binding and neutralizing antibody responses prior to challenge correlated with protection against viral replication postchallenge. These data are consistent with data with this vaccine from the phase 3 clinical trial.

## Introduction

SK bioscience in collaboration with GlaxoSmithKline (GSK) has developed a recombinant protein vaccine for COVID-19. The vaccine consists of GBP510 with the AS03 adjuvant. GBP510 is a self-assembling two-component protein nanoparticle, which displays 60 copies of the receptor binding domain (RBD) of SARS-CoV-2 Spike^1^. The nanoparticle component allows for the high-level multimeric display of the RBD antigen that enhances humoral immune responses even at lower doses^1–4^. This platform also allows for simple and efficient adjustment of the vaccine to match variant strains of SARS-CoV-2 or to match other emergent pathogens by swapping out the target antigen, which the nanoparticle displays^3^. Target antigens tested in this study were the ancestral strain matched RBD decorated nanoparticle (GBP510-Wu) and the B.1.351 strain (Beta) matched RBD decorated nanoparticle (GBP510-SA) as a booster. The AS03 Adjuvant System from GSK contains DL-alpha-tocopherol, squalene, and polysorbate 80 in an oil-in-water emulsion, which has been shown to enhance vaccine responses to influenza vaccines via innate immune system activation and increased antigen uptake^5,6^. The inclusion of AS03 may allow for the use of lower doses of protein antigen^6^.

A phase 1/2 clinical trial studied the immunogenicity of two doses, four weeks apart, of GBP510 with or without AS03^7^. The data from this early phase trial indicated that two 25 µg doses of GBP510 with AS03 was optimal and was assessed in a phase 3 trial^7^. The GBP510/AS03 vaccine, known as SKYCovione^TM^, has recently been approved in South Korea based on interim data from the phase 3 clinical trial comparing SKYCovione^TM^ to Vaxzevria, AstraZeneca’s ChAdOx-1-based COVID-19 vaccine (NCT05007951)^8–10^. This phase 3 trial was conducted to demonstrate the superiority of the ratio of positive to negative post-vaccination geometric mean neutralizing titers among participants compared to an approved vaccine and non-inferiority of the difference of seroconversion rates, measured by neutralizing antibody titer, also in comparison to an approved vaccine, rather than efficacy compared to a placebo group. The main reported readouts of this trial have been neutralizing antibody (NAb) titers, considered to be potential correlates of protection against SARS-CoV-2 infection^11–13^. It was demonstrated that the post-vaccination geometric mean titer of SKYCovione^TM^ was superior to Vaxzevria and the seroconversion rate of SKYCovione^TM^ in neutralizing antibody responses was non-inferior to Vaxzevria^9^.

We describe here the results of a study evaluating the immunogenicity of the GBP510/AS03 vaccine and protective efficacy against a high-dose SARS-CoV-2 Delta (B.1.617.2) challenge in rhesus macaques. This vaccine induced robust humoral immune responses after two doses and a greater response after three doses. A third dose of a Beta vaccine was comparable with a third dose of the ancestral strain vaccine in terms of binding and neutralizing antibody titers and protection. In all vaccinated groups, animals rapidly controlled viral replication from their lungs and upper airways following the challenge, with optimal protection with three vaccine doses.

## Results

### Study design

**Fig. 1:**
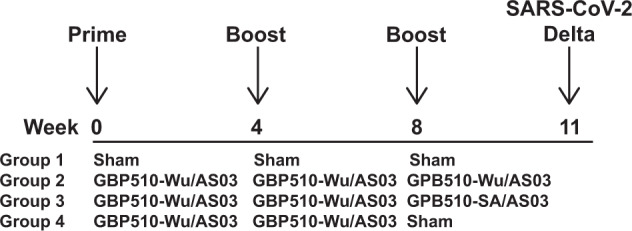
Vaccination and challenge schedule. Schematic of vaccine groups with the timing of immunization and challenge shown.

To evaluate the immunogenicity and protective efficacy of GBP510/AS03, we immunized 28 rhesus macaques with three sham doses (Group 1, *N* = 10), three doses of GBP510-Wu/AS03 at weeks 0, 4, and 8 (Group 2, *N* = 6), two doses of GBP510-Wu/AS03 at week 0 and 4 and a third dose of GBP510-SA/AS03 at week 8 (Group 3, *N* = 6), or two doses of GBP510-Wu/AS03 at weeks 0 and 4 only (Group 4, *N* = 6) (Fig. 1). Vaccines were administered by the intramuscular route, and each vaccine dose consisted of 25 µg of protein antigen in 500 µL with AS03 (Fig. 1). At week 11, all animals were challenged by the intranasal and intratracheal routes with 5 × 10^5^ TCID50 SARS-CoV-2 Delta (B.1.617.2) (Fig. 1).

### Vaccine immunogenicity

**Fig. 2:**
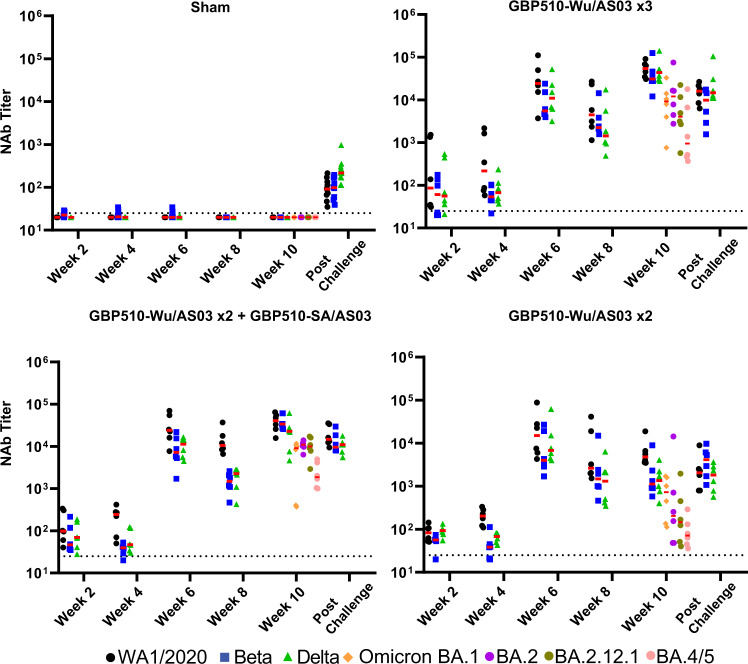
Neutralizing antibody responses measured to the vaccines. Neutralizing antibody (NAb) titers by luciferase-based pseudovirus neutralization assay for WA1/2020 (black circles), Beta (blue squares), and Delta (green triangles) matched pseudoviruses. The limit of quantitation (NT50 = 20) is represented by a dotted line. Medians are shown with red bars. Group 1 received three sham doses, Group 2 received three doses of GBP510-Wu, Group 3 received two doses of GBP510-Wu and one dose of GBP510-SA, and Group 4 received two doses of GBH510-Wu and one sham dose.

We evaluated serum neutralizing antibody (NAb) responses with lentiviral luciferase-based pseudoviral neutralization assays^14^, and binding antibody responses by RBD-specific ELISAs and RBD- and Spike (S)-specific electrochemiluminescence assays (ECLAs)^15^. All vaccinated animals in Groups 2-4 developed NAbs by two weeks after the first dose (Fig. 2). At week 6, two weeks after the second dose, all animals in Groups 2–4 developed high NAb titers against WA1/2020, Beta, and Delta. Median NAb titer in animals in Group 2 were 24,459, 5526, and 10,951 against WA1/2020, Beta, and Delta respectively. Median WA1/2020, Beta, and Delta NAb titers in animals in Group 3 were 23,906, 7229, and 11,266, and in Group 4 were 15,026, 4008, and 6744, respectively. At week 10, two weeks after the third dose in Group 2 and Group 3, median WA1/2020, Beta, and Delta NAb titers increased to 54,553, 30,967, and 43,878 in animals in Group 2 and to 40,618, 33,856, and 22,729 in animals in Group 3, respectively. Omicron BA.1, BA.2, BA.2.12.1, and BA.4/5 NAb titers were assayed for the week 10 timepoint, two weeks following the final immunizations. Median NAb titers to Omicron BA.1 were 9230, 9096, and 729 in Groups 2, 3, and 4 respectively. For BA.2 the median NAb titers were 12,034, 10984, and 205 in Groups 2, 3, and 4 respectively. BA.2.12.1 median NAb titers were 4044, 9964, and 146 for Groups 2, 3, and 4 respectively. Median NAb titers to BA.4/5 were 949, 1864, and 72 for Groups 2, 3, and 4 respectively. Sham animals in Group 1 showed no detectable NAbs prior to the challenge (Fig. 2).

**Fig. 3:**
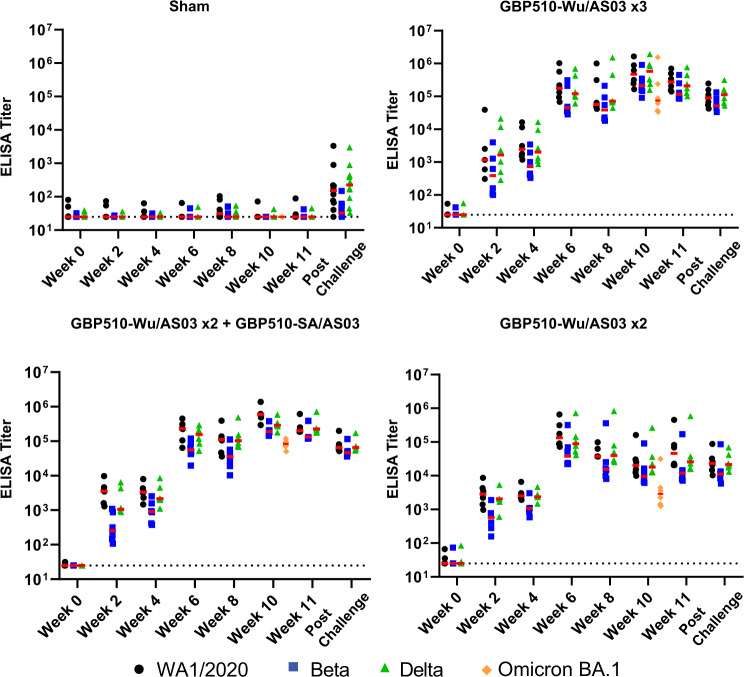
Binding antibody responses measured to the vaccines. Antibody binding titers enzyme-linked immunosorbent assay (ELISA) for WA1/2020 RBD (black circles), Beta RBD (blue squares), and Delta RBD (green triangles). The limit of quantitation (EPT = 25) is represented by a dotted line. Medians are shown with red bars. Group 1 received three sham doses, Group 2 received three doses of GBP510-Wu, Group 3 received two doses of GBP510-Wu and one dose of GBP510-SA, and Group 4 received two doses of GBH510-Wu and one sham dose.

Binding antibody responses by RBD-specific ELISA data were consistent with the NAb data and were observed in all vaccinated animals against WA1/2020, Beta, and Delta RBD (Fig. 3). Binding antibody titers were high against the ancestral RBD, slightly lower or comparable against Delta RBD, and lower against Beta RBD. Binding antibody responses increased following each vaccine dose, similar to NAb responses. Omicron BA.1 responses were similarly assayed at week 10 and, as with NAbs, were reduced compared to ancestral or other variants in each vaccinated group. RBD- and S-specific electrochemiluminescence assays (ECLA) data showed a similar pattern of high binding antibody titers to the ancestral and variant RBD (Fig. S1) and Spike proteins (Fig. S2).

Binding and neutralizing antibody responses induced by the vaccine waned markedly in the absence of a third boost as seen in Group 4 (Figs. 2 and 3). The limited time period between the third dose and the challenge did not allow for an assessment of the impact of waning immunity on protective efficacy. Moreover, Spike-specific IFNγ enzyme-linked immunospot (ELISPOT) responses were undetectable in all vaccinated animals (data not shown).

### Protective efficacy

**Fig. 4:**
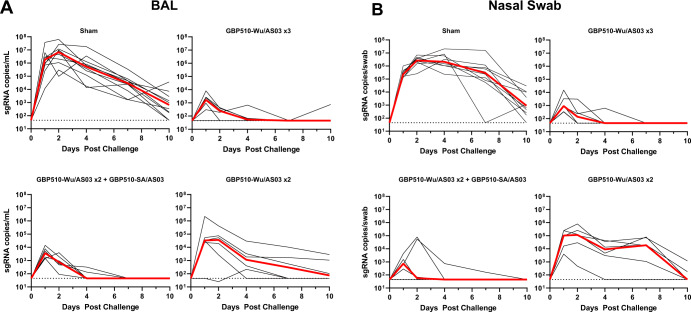
Viral loads following challenge with delta SARS-CoV-2. Group 1 received three sham doses, Group 2 received three doses of GBP510-Wu, Group 3 received two doses of GBP510-Wu and one dose of GBP510-SA, and Group 4 received two doses of GBH510-Wu and one sham dose. **A** Subgenomic RNA (sgRNA) copies/mL in the bronchoalveolar lavage (BAL) following Delta SARS-CoV-2 challenge days 0 to 10. Medians are shown with red lines. **B** Subgenomic RNA (sgRNA) copies/swab in the nasal swabs (NS) following Delta SARS-CoV-2 challenge days 0 to 10. Medians are shown with red lines.

At week 11, all animals were challenged with 5×10^5^ TCID50 SARS-CoV-2 B.1.617.2 (Delta) by the intranasal and intratracheal routes. Productive infection was expected in all animals given the mismatch between the vaccine and challenge strains and the high dose challenge. Viral loads were assessed in the bronchoalveolar lavage (BAL) and nasal swabs (NS) of the animals by subgenomic E RNA (sgRNA) specific quantitative RT-PCR after challenge (Fig. 4A, B)^16^.

**Fig. 5:**
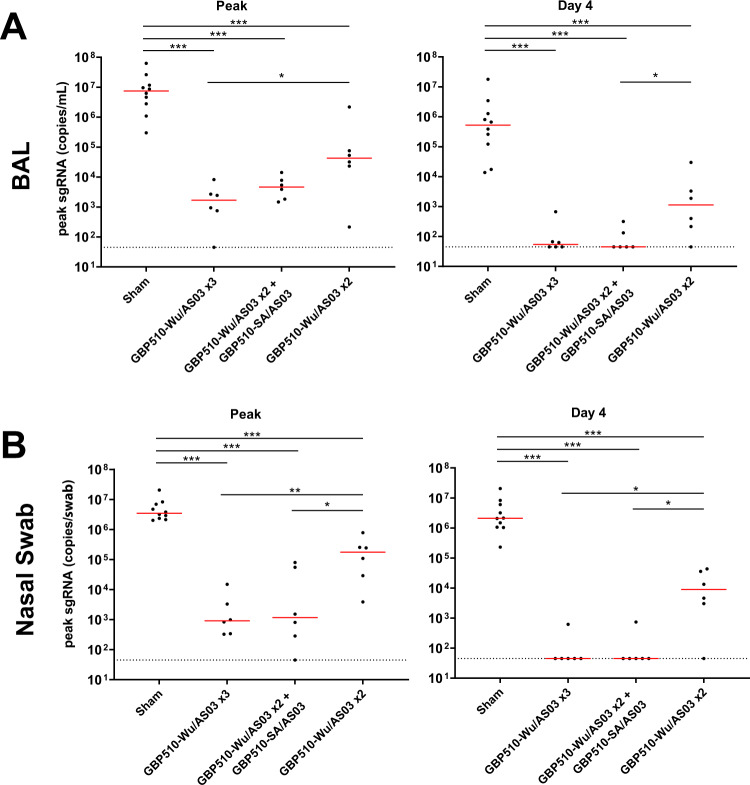
Peak and day 4 viral loads following challenge with delta SARS-CoV-2. Group 1 received three sham doses, Group 2 received three doses of GBP510-Wu, Group 3 received two doses of GBP510-Wu and one dose of GBP510-SA, and Group 4 received two doses of GBH510-Wu and one sham dose. **A** Subgenomic RNA (sgRNA) copies/mL in the bronchoalveolar lavage (BAL) following Delta SARS-CoV-2 challenge at peak and at Day 4 postchallenge. Median lines are shown with red bars. Vaccinated groups were compared with sham controls by two-sided Mann-Whitney tests. ***p < 0.0005, **p < 0.005, *p < 0.05. **B** Subgenomic RNA (sgRNA) copies/swab in the nasal swabs (NS) following Delta SARS-CoV-2 challenge at peak and at Day 4 postchallenge. Median lines are shown with red bars. Vaccinated groups were compared with sham controls by two-sided Mann-Whitney tests. ***p < 0.0005, **p < 0.005, *p < 0.05.

The median peak sgRNA level in BAL of the sham animals was 6.87 log RNA copies/mL (Fig. 5A). In triple GBP510-Wu/AS03 vaccinated animals, the median peak sgRNA level was lower at 3.22 log copies/mL in BAL, demonstrating a 3.65 log reduction (*P* = 0.0002, two-sided Mann-Whitney test). Similarly, in animals that received two doses of GBP510-Wu/AS03 followed by a dose of GBP510-SA/AS03, the median peak sgRNA levels were 3.66 log copies/mL in BAL, a 3.21 log reduction (*P* = 0.0002, two-sided Mann-Whitney test). Animals that received only two doses of GBP510-Wu/AS03 and no third dose had peak sgRNA levels of 4.62 log copies/mL in BAL, which was lower than the sham animals (*P* = 0.0010, two-sided Mann-Whitney test) but higher than the animals that received three doses of GBP510-Wu/AS03 (*P* = 0.0411, two-sided Mann-Whitney test). All vaccine regimens led to significantly lower median BAL sgRNA levels by day 4 postchallenge (*P* = 0.0002, *P* = 0.0002, and *P* = 0.0010 for Groups 2-4 respectively, two-sided Mann-Whitney test). Three vaccine doses of GBP510-Wu/AS03 or GBP510-Wu/AS03 and GBP510-SA/AS03 led to 3.98 or 4.06 log reductions of median viral loads in BAL, respectively, on day 4. Two GBP510-Wu/AS03 doses led to a lesser, though still substantial, 2.66 log reduction in median day 4 BAL viral loads.

In nasal swabs, the median peak sgRNA levels of the sham animals were 6.54 log RNA copies/swab (Fig. 5B). The median peak for sgRNA in the NS of the triple GBP510-Wu/AS03, double GBP510-Wu/AS03 plus GBP510-SA/AS03, and double GBP510-Wu/AS03 vaccinated animals were 2.95, 3.06, and 5.24 log copies/swab respectively, all significantly lower than the sham animals (*P* = 0.0002, two-sided Mann-Whitney test). These values represent 3.59, 3.48, and 1.3 log reductions in sgRNA in NS, respectively. Median peak sgRNA levels in the NS were significantly higher for the animals that received two doses of GBP510-Wu/AS03 compared to those that received three (*P* = 0.0043, two-sided Mann-Whitney test) or two and a dose of GBP510-SA (*P* = 0.0260, two-sided Mann-Whitney test). On day 4 postchallenge, all vaccinated animals had significantly reduced median sgRNA levels in the NS (*P* = 0.0002, two-sided Mann-Whitney test). The three-dose regimens led to 4.67 log reductions in median viral loads by day 4, and the two-dose regimen led to a 2.37 log reduction. None of the vaccine regimens protected against the acquisition of infection in this heterologous challenge model.

Lung histopathology scores were significantly lower in animals, which received all vaccine regimens compared with sham animals; *P* = 0.0173, *P* = 0.0002, *P* = 0.0483 for Groups 2, 3, and 4 respectively (Kruskal-Wallis test) (fig. S3).

### Immune correlates of protection

**Fig. 6:**
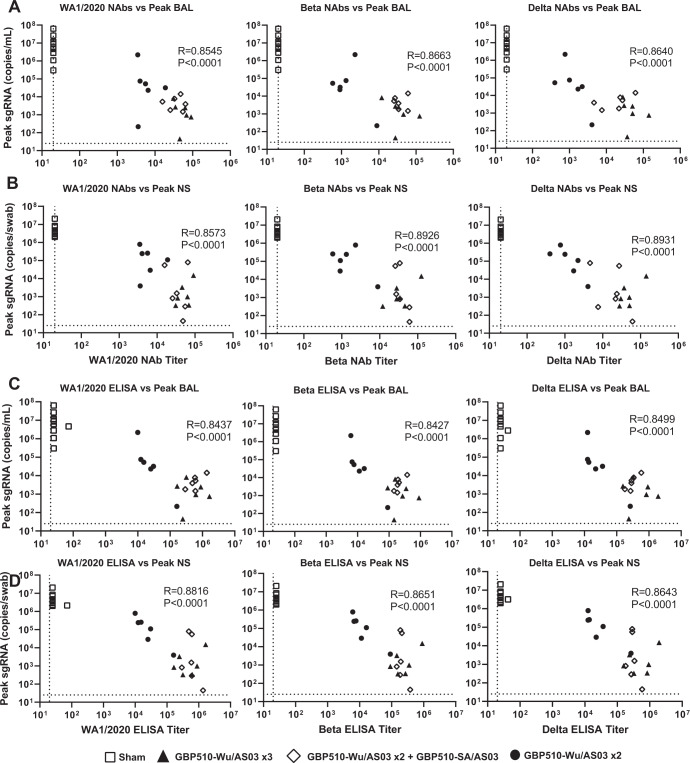
Correlates of humoral responses and viral loads. Group 1 (open squares) received three sham doses, Group 2 (solid triangles) received three doses of GBP510-Wu, Group 3 (open diamonds) received two doses of GBP510-Wu and one dose of GBP510-SA, and Group 4 (solid circles) received two doses of GBH510-Wu and one sham dose. **A** The correlation between week 10 NAbs against WA1/2020 (*R* = 0.8545, *P* < 0.0001, two-sided Spearman rank-correlation tests), Beta *(R* = 0.8663, *P* < 0.0001, two-sided Spearman rank-correlation tests), and Delta (*R* = 0.8640, *P* < 0.0001, two-sided Spearman rank-correlation tests) vs the peak viral loads in the BAL after challenge with Delta SARS-CoV-2. **B** The correlation between week 10 NAbs against WA1/2020 (*R* = 0.8573, *P* < 0.0001, two-sided Spearman rank-correlation tests), Beta (*R* = 0.8026, *P* < 0.0001, two-sided Spearman rank-correlation tests), and Delta (*R* = 0.8931, *P* < 0.0001, two-sided Spearman rank-correlation tests) vs the peak viral loads in the NS after challenge with Delta SARS-CoV-2. **C** The correlation between week 10 RBD specific binding antibodies against WA1/2020 (*R* = 0.8437, *P* < 0.0001, two-sided Spearman rank-correlation tests), Beta (*R* = 0.8427, *P* < 0.0001, two-sided Spearman rank-correlation tests), and Delta (*R* = 0.8499, *P* < 0.0001, two-sided Spearman rank-correlation tests) vs the peak viral loads in the BAL after challenge with Delta SARS-CoV-2. **D** The correlation between week 10 RBD specific binding antibodies against WA1/2020 (*R* = 0.8616, *P* < 0.0001, two-sided Spearman rank-correlation tests), Beta (*R* = 0.8651, *P* < 0.0001, two-sided Spearman rank-correlation tests), and Delta (*R* = 0.8641, *P* < 0.0001, two-sided Spearman rank-correlation tests) vs the peak viral loads in the NS after challenge with Delta SARS-CoV-2.x.

The size of this study and the degree of variability in vaccine responses and viral loads allowed for an immune correlate analysis. Peak viral loads in BAL correlated inversely with week 10 NAb titers immediately pre-challenge against the ancestral WA1/2020, Beta, and Delta strains (*P* < 0.0001 *R* = 0.8545, *P* < 0.0001 *R* = 0.8663, and *P* < 0.0001 *R* = 0.8640 respectively, two-sided Spearman rank-correlation tests) (Fig. 6A). The same correlation pattern is seen for the week 10 NAb titers and the peak viral loads in NS (Fig. 6B). The week 10 ELISA titers to WA1/2020 RBD, Beta RBD, and Delta RBD also correlated inversely with the peak viral loads in BAL postchallenge (*P* < 0.0001 *R* = 0.8437, *P* < 0.0001 *R* = 0.8427, and *P* < 0.0001 *R* = 0.8499 respectively, two-sided Spearman rank-correlation test) (Fig. 6C) and in NS (Fig. 6D). These data suggest that antibody responses elicited by GBP510/AS03 correlated with protective efficacy. The long-term correlates of durability were not assessed in this study.

## Discussion

The development of additional safe and efficacious vaccines against SARS-CoV-2 continues to be a global priority. Our data demonstrate the immunogenicity and protective efficacy of the GBP510/AS03 vaccine against challenge with SARS-CoV-2 Delta in rhesus macaques. Three-dose regimens were more immunogenic and protective than the two-dose regimen, although differences in efficacy may also reflect different times from the final immunization to the challenge.

GBP510-Wu adjuvanted with AS03, also known as SKYCovione^TM^, has been tested as a two-dose regimen in a phase 3 clinical trial in comparison to AstraZeneca’s Vaxzevria. SKYCovione^TM^ induced a 2.93 times greater NAb response compared with Vaxzevria two weeks following the second dose^9^. These data recently led to marketing authorization for SKYCovione^TM^ with the Republic of Korea’s Ministry of Food and Drug Safety^10^.

The inclusion of a GBP510-Beta variant booster did not substantially alter the profile of NAb responses compared with the GBP510-Wu booster, in part because a third dose induced NAb responses against multiple variants^17–20^.

Recombinant protein-based vaccines have been used historically and recently often with great success to induce strong and often protective humoral immune responses^21–23^. Many recombinant protein vaccine candidates against SARS-CoV-2, including GBP510/AS03 from SK bioscience, NVX-CoV2373 from Novavax, and CoV2 preS dTM from Sanofi among others, have been tested in pre-clinical and clinical settings^23–25^. Novavax reported similarly high rates of seroconversion to SK bioscience. In the Novavax placebo-controlled Phase 3 trial which began in December 2020, Novavax reported high efficacy: 100% prevention of moderate-to-severe disease against primarily the Alpha strain^24,25^. The Novavax vaccine also reported the induction of antigen-specific CD4 + T cell responses^26,27^.

Most of these protein vaccines aim to induce robust humoral responses via adjuvanted protein, specifically using either full-length, trimeric Spike as in the Sanofi and Novavax vaccines, or using RBD that is otherwise multimerized^23^. The nanoparticle display utilized in GBP510/AS03 facilitates the display of 60 RBD copies per nanoparticle, greatly increasing the avidity of the antigen and potentially increasing uptake by antigen-presenting cells, lymph node trafficking, and B cell activation^1,28,29^.

GBP510/AS03 induced potent neutralizing and binding antibody responses in rhesus macaques against ancestral and variant strains including Omicron BA.1. Antibody responses, both neutralizing and otherwise, have been correlated with protection against the acquisition of SARS-CoV-2 and development of COVID-19 disease^12,30–32^. Neutralizing antibodies specifically have been shown to correlate strongly with protective efficacy, however, it has also been found that non- or pre-neutralizing antibody responses are also protective and that Th1 and CD8 T cell responses may also play roles in protection^30,32–34^. Compared with previous reports of antibody responses induced by mRNA and Ad26-based COVID-19 vaccines in macaques, the binding and neutralizing antibody responses induced by GBP510/AS03 were greater in magnitude^33,35–37^, although mRNA and Ad26 vaccines also induced T cell responses^33,38^.

GBP510/AS03 vaccination was shown here to be highly protective against challenge with Delta SARS-CoV-2 in rhesus macaques, correlating with antibody responses. Neutralizing antibody responses have been considered a correlate of protection for other COVID-19 vaccines, but further research of local immune responses is warranted^11,12,31,39^. In particular, future research should focus on the induction of mucosal antibody responses^40–44^ as well as resident cellular responses^40–45^.

In summary, we show that the GBP510/AS03 vaccine induces high antibody titers against multiple SARS-CoV-2 variants and provides robust protection against a high-dose, heterologous Delta challenge in macaques, although we observed waning immunity and did not detect cellular immune responses to this vaccine. These data, along with the phase 3 human data, supports the use of SKYCovione^TM^ for the prevention of COVID-19^7^.

## Methods

### Animals and study design

28 outbred adult male and female rhesus macaques ages 4–6 years old were randomly allocated to 4 experimental groups (*N* = 6/vaccine group and *N* = 10 for the sham). All animals were singly housed at Bioqual, Inc. (Rockville, MD). Groups of animals were immunized with 25 µg of GBP510-Wu with AS03 (GSK) or sham at week 0 and week 4. At week 8 one group (Group 2) received an additional dose of 25 µg of GBP510-Wu with AS03, one group (Group 3) received a 25 µg dose of GBP510-SA with AS03, and the other two groups (Group 1 and 4) received sham immunizations. At week 11, all animals were challenged with 5 × 10^5^ TCID50 SARS-CoV-2 B.1.617.2 (Delta) by the intranasal and intratracheal routes in a total volume of 2 mL. Following the challenge, viral loads were assessed in bronchoalveolar lavage (BAL) and nasal swab (NS) samples by RT-PCR for E subgenomic RNA (sgRNA). Immunologic and virologic assays were performed blinded. All animal studies were conducted in compliance with all relevant local, state, and federal regulations and were approved by the Bioqual Institutional Animal Care and Use Committee (IACUC).

### Pseudovirus neutralizing antibody assay

The SARS-CoV-2 pseudoviruses expressing a luciferase reporter gene were used to measure pseudovirus-neutralizing antibodies^14^. In brief, the packaging construct psPAX2 (AIDS Resource and Reagent Program), luciferase reporter plasmid pLenti-CMV Puro-Luc (Addgene) and spike protein expressing pcDNA3.1-SARS-CoV-2 SΔCT were co-transfected into HEK293T cells (ATCC CRL_3216) with lipofectamine 2000 (ThermoFisher Scientific). Pseudoviruses of SARS-CoV-2 variants were generated by using WA1/2020 strain (Wuhan/WIV04/2019, GISAID accession ID: EPI_ISL_402124), B.1.617.2 (Delta, GISAID accession ID: EPI_ISL_2020950), or B.1.1.529 (Omicron, GISAID ID: EPI_ISL_7358094.2). The supernatants containing the pseudotype viruses were collected 48 h after transfection; pseudotype viruses were purified by filtration with 0.45-μm filter. To determine the neutralization activity of human serum, HEK293T-hACE2 cells were seeded in 96-well tissue culture plates at a density of 2.0 × 10^4^ cells per well overnight. Three-fold serial dilutions of heat-inactivated serum samples were prepared and mixed with 50 μl of pseudovirus. The mixture was incubated at 37 °C for 1 h before adding to HEK293T-hACE2 cells. After 48 h, cells were lysed in Steady-Glo Luciferase Assay (Promega) according to the manufacturer’s instructions. SARS-CoV-2 neutralization titers were defined as the sample dilution at which a 50% reduction (NT50) in relative light units was observed relative to the average of the virus control wells.

### Enzyme-linked immunosorbent assay (ELISA)

SARS-CoV-2 spike receptor-binding domain (RBD)-specific binding antibodies in serum were assessed by ELISA. 96-well plates were coated with 1 μg/mL of similarly produced SARS-CoV-2 WA1/2020, B.1.617.2 (Delta), or B.1.1.529 (Omicron) RBD protein in 1× Dulbecco phosphate-buffered saline (DPBS) and incubated at 4 °C overnight. Assay performance was similar for these four RBD proteins. After incubation, plates were washed once with wash buffer (0.05% Tween 20 in 1× DPBS) and blocked with 350 μL of casein block solution per well for 2 to 3 h at room temperature. Following incubation, the block solution was discarded, and the plates were blotted dry. Serial dilutions of heat-inactivated serum diluted in Casein block were added to wells, and plates were incubated for 1 hour at room temperature, prior to 3 more washes and a 1-hour incubation with a 1 μg/mL dilution of anti–macaque IgG horseradish peroxidase (HRP) (Nonhuman Primate Reagent Resource, AB_2819289) at room temperature in the dark. Plates were washed 3 times, and 100 μL of SeraCare KPL TMB SureBlue Start solution was added to each well; plate development was halted by adding 100 μL of SeraCare KPL TMB Stop solution per well. The absorbance at 450 nm was recorded with a VersaMax microplate reader (Molecular Devices). For each sample, the ELISA endpoint titer was calculated using a 4-parameter logistic curve fit to calculate the reciprocal serum dilution that yields an absorbance value of 0.2, twice the average absorbance of naïve serum. The raw OD values were transferred into GraphPad Prism for analysis. A standard curve was interpolated using a sigmoidal four-parameter logistic (4PL) fit. To quantify the endpoint titer, the interpolation function was used to calculate the dilution at which the OD value would be equal to a value of 0.2.

### Electrochemiluminescence assay (ECLA)

ECLA plates (MesoScale Discovery SARS-CoV-2 IgG, Panels 22, 23) were designed and produced for multiplex binding assays with up to 10 antigen spots in each well, including either Spike or RBD proteins from multiple SARS-CoV-2 variants^15^. The plates were blocked with 150 µL of Blocker A (1% BSA in distilled water) solution for at least 30 minutes at room temperature shaking at 700 rpm with a digital microplate shaker. During blocking the serum was diluted to 1:5000 or 1:50,000 in Diluent 100. The calibrator curve was prepared by diluting the calibrator mixture from MSD 1:10 in Diluent 100 and then preparing a 7-step 4-fold dilution series plus a blank containing only Diluent 100. The plates were then washed 3 times with 150 μL of Wash Buffer (0.5% Tween in 1x PBS), blotted dry, and 50 μL of the diluted samples and calibration curve were added in duplicate to the plates and set to shake at 700 rpm at room temperature for at least 2 h. The plates were again washed 3 times and 50 μL of SULFO-Tagged anti-Human IgG detection antibody diluted to 1x in Diluent 100 was added to each well and incubated shaking at 700 rpm at room temperature for at least 1 h. Plates were then washed 3 times and 150 μL of MSD GOLD Read Buffer B was added to each well and the plates were read immediately after on a MESO QuickPlex SQ 120 machine. MSD titers for each sample was reported as Relative Light Units (RLU) which were calculated as Sample RLU minus Blank RLU and then fit using a logarithmic fit to the standard curve. The upper limit of detection was defined as 2×10^6^ RLU for each assay and the signal for samples, which exceeded this value at 1:5,000 serum dilution was run again at 1:50,000 and the fitted RLU was multiplied by 10 before reporting. The lower limit of detection was defined as 1 RLU and an RLU value of 100 was defined to be positive for each assay.

### Subgenomic RT-PCR assay

SARS-CoV-2 E gene subgenomic RNA (sgRNA) was assessed by RT-PCR using specific primers and probes^16^. A standard was generated by first synthesizing a gene fragment of the subgenomic E gene. The gene fragment was subsequently cloned into a pcDNA3.1+ expression plasmid using restriction site cloning (Integrated DNA Technologies). The insert was in vitro transcribed to RNA using the AmpliCap-Max T7 High Yield Message Maker Kit (CellScript). Log dilutions of the standard were prepared for RT-PCR assays ranging from 1×10^10^ copies to 1×10^−1^ copies. Viral loads were quantified from bronchoalveolar lavage (BAL) fluid and nasal swabs (NS). RNA extraction was performed on a QIAcube HT using the IndiSpin QIAcube HT Pathogen Kit according to manufacturer’s specifications (Qiagen). The standard dilutions and extracted RNA samples were reverse transcribed using SuperScript VILO Master Mix (Invitrogen) following the cycling conditions described by the manufacturer. A Taqman custom gene expression assay (Thermo Fisher Scientific) was designed using the sequences targeting the E gene sgRNA. The sequences for the custom assay were as follows, forward primer, sgLeadCoV2.Fwd: CGATCTCTTGTAGATCTGTTCTC, E_Sarbeco_R: ATATTGCAGCAGTACGCACACA, E_Sarbeco_P1 (probe): VIC-ACACTAGCCATCCTTACTGCGCTTCG-MGBNFQ. Reactions were carried out in duplicate for samples and standards on the QuantStudio 6 and 7 Flex Real-Time PCR Systems (Applied Biosystems) with the thermal cycling conditions: initial denaturation at 95 °C for 20 s, then 45 cycles of 95 °C for 1 s and 60 °C for 20 s. Standard curves were used to calculate subgenomic RNA copies per ml or per swab. The quantitative assay sensitivity was determined as 50 copies per ml or per swab.

### Histopathology

Representative tissue sections were collected from the right and left lung lobes from each animal at necropsy. All tissues were fixed in 10% formalin and block-sectioned at 5 µm. The slides were baked for 30–60 min at 65 °C, deparaffinized in xylene, rehydrated through a series of graded ethanol to distilled water and then stained with hematoxylin and eosin. Blinded histopathological evaluation was performed by a board-certified veterinary pathologist (A.J.M.). Two hematoxylin and eosin (H&E) sections were evaluated and scored for lesions including interstitial inflammation, endothelialitis, and Type-II pneumocyte hyperplasia as previously described^46^.

### Statistical analyses

Descriptive statistics were performed using GraphPad Prism 9.1.2, (GraphPad Software, San Diego, California). Virologic data were generated in duplicate and were compared by two-sided Mann-Whitney tests with Bonferroni corrections for multiple comparisons. Correlations were tested with two-sided Spearman rank-correlation tests. Lung histopathology scores were compared by Kruskal-Wallis test. *P* values less than 0.05 were considered significant.

### Reporting summary

Further information on research design is available in the Nature Research Reporting Summary linked to this article.

## Supplementary information

Supplemental Information

REPORTING SUMMARY

## Data Availability

All data are available in the manuscript or the supplementary material. Correspondence and requests for materials should be addressed to D.H.B. (dbarouch@bidmc.harvard.edu).
