# Supplementary material for: Immunogenicity and protective efficacy of GBP510/AS03 vaccine against SARS-CoV-2 delta challenge in rhesus macaques
Source: NPJ Vaccines. 2023 Feb 23;8:23. doi: 10.1038/s41541-023-00622-0 (PMC9947939; doi:10.1038/s41541-023-00622-0)
Supplement: Supplementary file 1 — Supplemental Information [file 41541_2023_622_MOESM1_ESM.pdf]

Supplementary Information

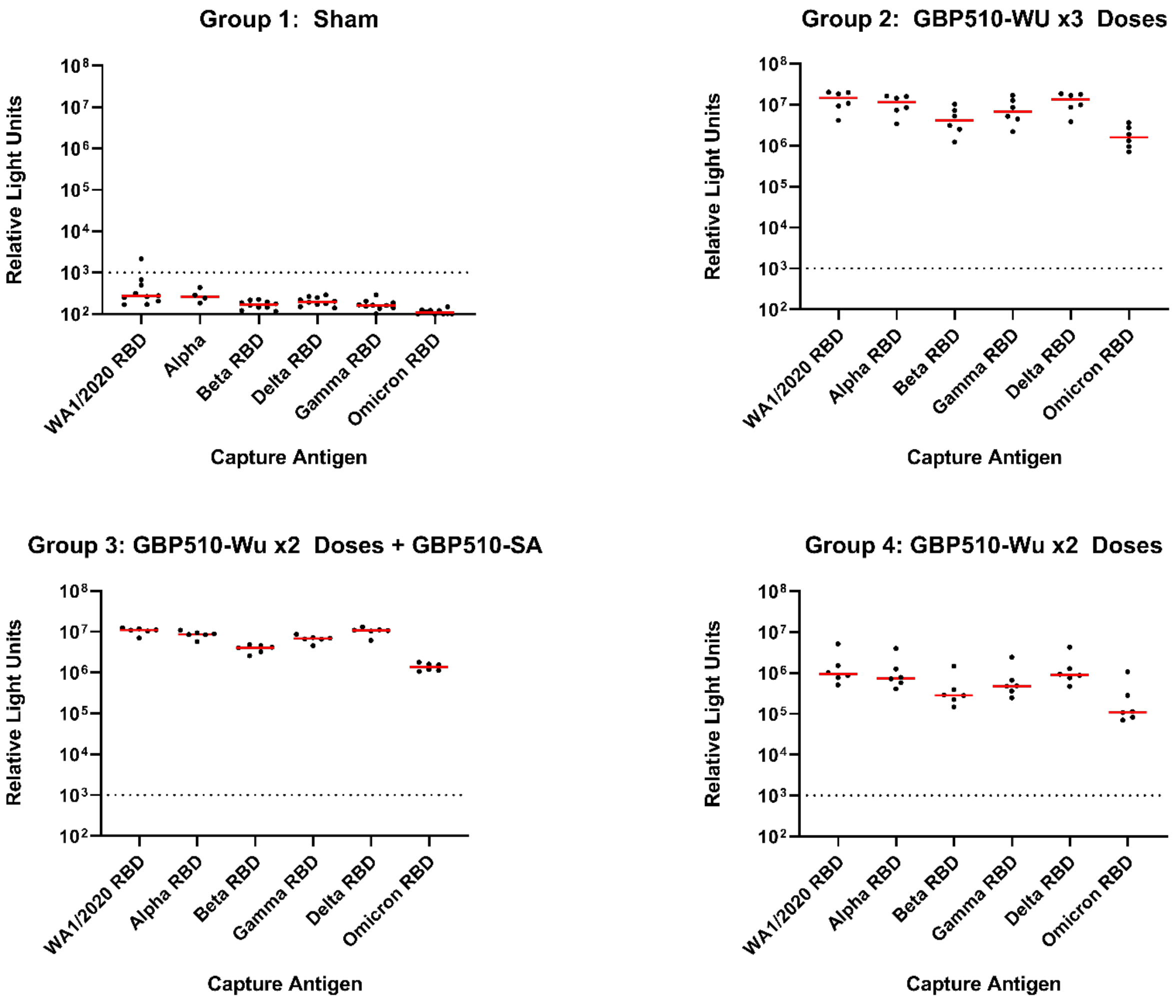

Supplementary Figure 1. Electro-chemiluminescent (ECLA) measured humoral responses to ancestral (WA1/2020) and variant strain RBDs including Alpha, Beta, Delta, Gamma, and Omicron (BA.1).

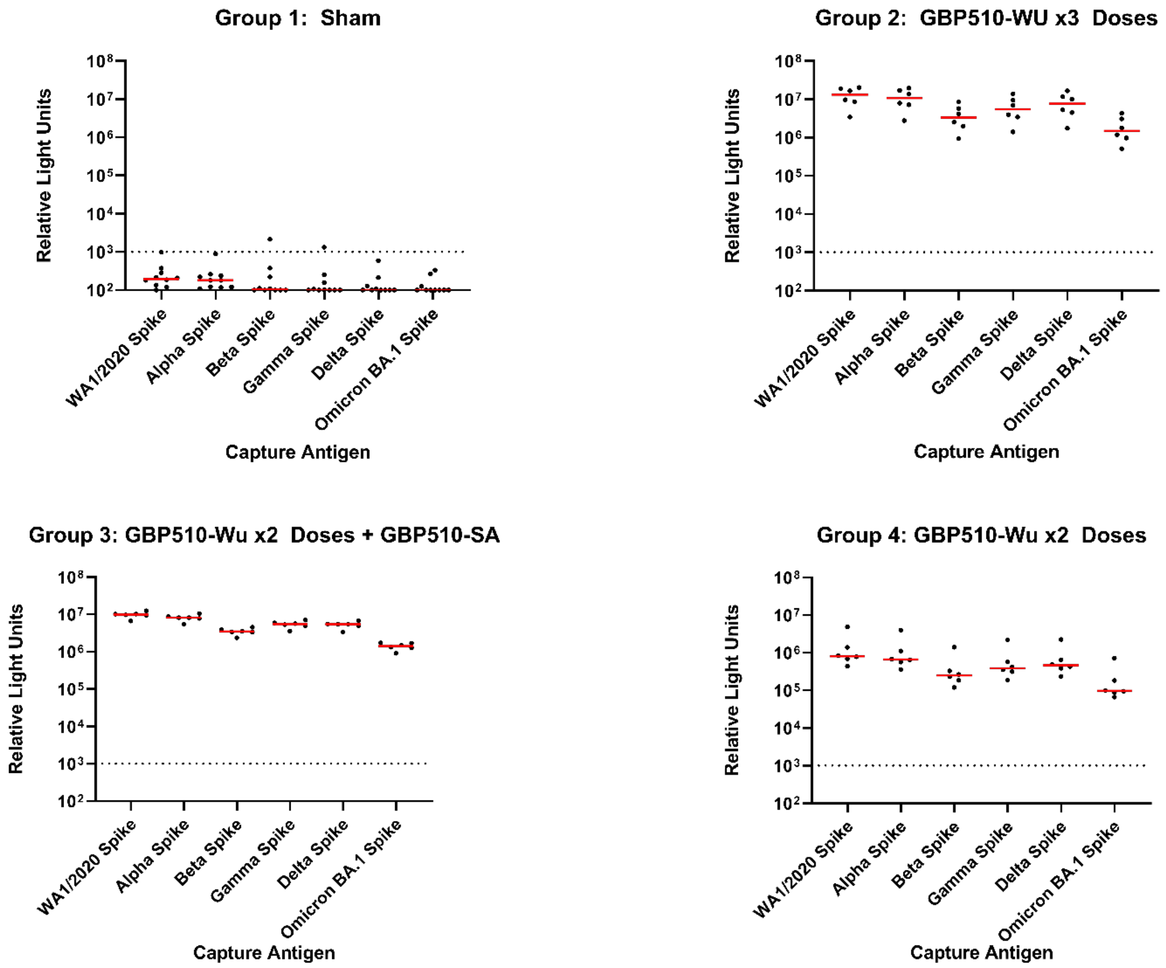

Supplementary Figure 2. Electro-chemiluminescent (ECLA) measured humoral responses to ancestral (WA1/2020) and variant strain Spikes including Alpha, Beta, Delta, Gamma, and Omicron (BA.1).

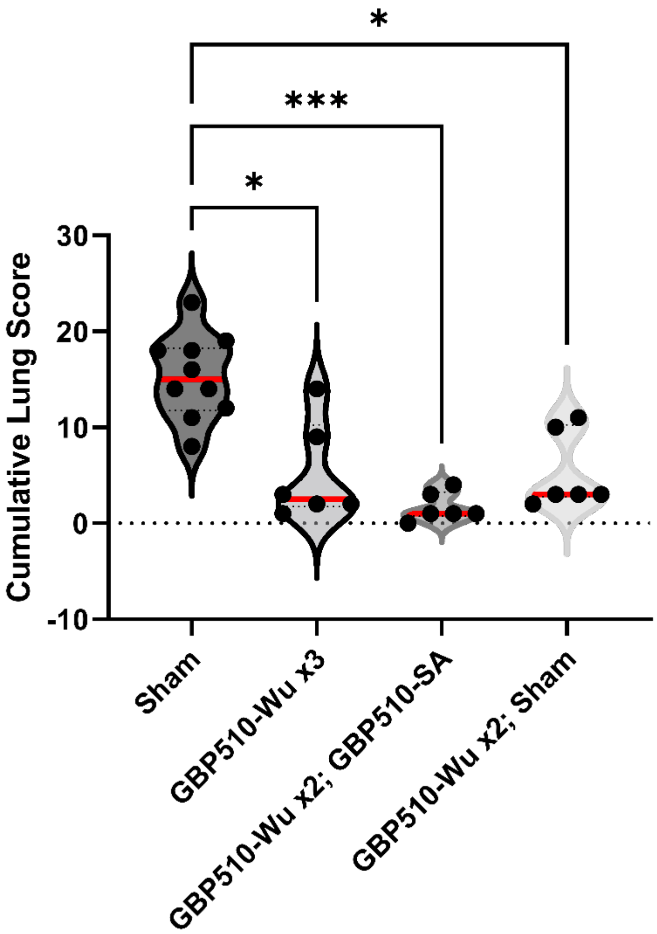

Supplementary Figure 3. Cumulative lung score via histopathology for each of the groups studied. Vaccinated groups were compared by Kruskal-Wallis test to the sham group, P=0.0173 for GBP510-Wu x3, P=0.0002 for GBP510-Wu x2; GBP510-SA, and P=0.0483 for GBP510-Wu x2.
